# Automated image registration of RGB, hyperspectral and chlorophyll fluorescence imaging data

**DOI:** 10.1186/s13007-024-01296-y

**Published:** 2024-11-17

**Authors:** Hans Lukas Bethge, Inga Weisheit, Mauritz Sandro Dortmund, Timm Landes, Miroslav Zabic, Marcus Linde, Thomas Debener, Dag Heinemann

**Affiliations:** 1 Institute of Horticultural Production Systems, Department of Phytophotonics, Leibniz University Hannover, Herrenhäuser Str. 2, 30419 Hannover, Germany; 2 Hannover Centre for Optical Technologies, Leibniz University Hannover, Nienburger Straße 17, 30167 Hannover, Germany; 3 Cluster of Excellence PhoenixD, Leibniz University Hannover, Welfengarten 1a, 30167 Hannover, Germany; 4 Institute of Plant Genetics, Department of Molecular Plant Breeding, Leibniz University Hannover, Herrenhäuser Str. 2, 30419 Hannover, Germany

**Keywords:** Multi-modal image registration, High-throughput phenotyping, RGB imaging, Hyperspectral imaging, Chlorophyll fluorescence, Sensor fusion, Affine transform

## Abstract

**Background:**

The early and specific detection of abiotic and biotic stresses, particularly their combinations, is a major challenge for maintaining and increasing plant productivity in sustainable agriculture under changing environmental conditions. Optical imaging techniques enable cost-efficient and non-destructive quantification of plant stress states. Monomodal detection of certain stressors is usually based on non-specific/indirect features and therefore is commonly limited in their cross-specificity to other stressors. The fusion of multi-domain sensor systems can provide more potentially discriminative features for machine learning models and potentially provide synergistic information to increase cross-specificity in plant disease detection when image data are fused at the pixel level.

**Results:**

In this study, we demonstrate successful multi-modal image registration of RGB, hyperspectral (HSI) and chlorophyll fluorescence (ChlF) kinetics data at the pixel level for high-throughput phenotyping of *A. thaliana* grown in Multi-well plates and an assay with detached leaf discs of *Rosa × hybrida* inoculated with the black spot disease-inducing fungus *Diplocarpon rosae*. Here, we showcase the effects of (i) selection of reference image selection, (ii) different registrations methods and (iii) frame selection on the performance of image registration via affine transform. In addition, we developed a combined approach for registration methods through NCC-based selection for each file, resulting in a robust and accurate approach that sacrifices computational time. Since image data encompass multiple objects, the initial coarse image registration using a global transformation matrix exhibited heterogeneity across different image regions. By employing an additional fine registration on the object-separated image data, we achieved a high overlap ratio. Specifically, for the *A. thaliana* test set, the overlap ratios (OR_Convex_) were 98.0 ± 2.3% for RGB-to-ChlF and 96.6 ± 4.2% for HSI-to-ChlF. For the *Rosa × hybrida* test set, the values were 98.9 ± 0.5% for RGB-to-ChlF and 98.3 ± 1.3% for HSI-to-ChlF.

**Conclusion:**

The presented multi-modal imaging pipeline enables high-throughput, high-dimensional phenotyping of different plant species with respect to various biotic or abiotic stressors. This paves the way for in-depth studies investigating the correlative relationships of the multi-domain data or the performance enhancement of machine learning models via multi modal image fusion.

**Supplementary Information:**

The online version contains supplementary material available at 10.1186/s13007-024-01296-y.

## Background

Early and specific detection of plant responses to abiotic and biotic stress factors, particularly their combinations, is a major challenge for maintaining and increasing plant productivity in precision agriculture [1]. In this context, precision agriculture attempts to selectively consider heterogeneous plant canopies through the perception of individual plant phenotypes [2]. The information content of common sensor-based phenotypic detection is therefore crucial for deriving a plant-specific recommendation for interactions/counteractive measures. In addition, phenotyping of plants is essential for the identification of stress-related genes by providing phenotypic data for the breeding of stress-resilient genotypes. Recent technological advances in genotyping have accelerated the demand for automation and precision in phenotyping, but the rate of progress in phenotyping has not kept pace, creating a bottleneck [3]. As part of the breeding process, a large number of different genotypes are exposed to various biotic and abiotic stress factors in order to quantitatively evaluate their yield and stress resilience [4]. In contrast to yield, some breeding-relevant traits can be tested at an early stage of plant development. Recently, Li et al. (2023) proposed a space-efficient culture system (PhenoWell^®^) [5] uniquely designed for high-throughput screening of various abiotic stress factors on the growth performance of *Arabidopsis thaliana* and *Zea mays*. This system allows for the rapid and efficient evaluation of stress responses, facilitating early-stage identification of resilient genotypes.

Optical imaging techniques enable cost-efficient and non-destructive quantification of the stress state of plants [6–8]. Monomodal detection of certain stressors is usually based on non-specific/indirect features and therefore is commonly limited in their cross-specificity to other stressors [9]. The fusion of multi-domain sensor systems can provide more discriminative features for machine learning models and potentially provide synergistic information to increase cross-specificity in plant disease detection [1, 10]. Multi-modal image registration is a promising tool for (i) fusing low-contrast but high-dimensional data with high-contrast but low-dimensional data to enable automated plant segmentation [11], (ii) enhancing the predictive performance of machine learning models by increasing the number of potentially discriminative features [10], and (iii) combining multi-domain data to develop new plant status proxies. To date, most research has focused on the development of stress proxies from single-sensor systems rather than making use of the benefits of a multi-sensor approach [1], likely due to the lack of an automated data processing pipeline, limited commercial multi-sensor systems, higher costs and limited practical applicability. Enhancing information content by multi-modal data acquisition could address limitations like low cross-specificity in phenotyping; but requires the superposition of the different modalities.

Multi-modal image registration in plant science ranges from 2D registration of thermal to RGB images by manual control points [12], 2D registration of thermal to RGB images by an automated registration of Canny edge-filtered images [13], 2D registration of thermal to RGB-D images by automated registration of edge-filtered images and feature-based detectors [14], 2D registration of fluorescence to RGB images by automated registration approach [11, 15, 16], 3D stereo registration of multispectral and NIR images [17] and 3D image registration of RGB-D, thermal and hyperspectral data by a ray casting approach [18]. The challenge in multi-modal image registration lies in the different representation of image scenes. This is particularly pronounced when key features show limited similarity, and direct correlations between image intensities are absent. To tackle these complexities, a comprehensive array of registration techniques has been established, encompassing phase correlation, feature-based approaches, and mutual information. Frequency-based methods such as phase correlation, transform both input images into the Fourier domain and estimate the transform by finding the global peak correlation of Fourier parameters, such as amplitude or phase information [19]. The phase-only correlation (POC) method [20] focuses exclusively on phase information and is therefore robust to intensity differences and noise, captured in the amplitude parameter. Feature-based methods attempt to identify key points such as edges, corners or gradients in the pixel neighborhoods of both images. These key points are then used to calculate the transformation matrix between the images through feature matching and filtering, for example, via the random sample consensus (RANSAC [21]), algorithm [22]. The enhanced correlation coefficient (ECC [23]), is a similarity metric, an extension of normalized cross correlation (NCC, Eq. 1), that can be interpreted as a measure of correlation between zero-mean and variance-normalized image values. Unlike the sum of squared differences (SSD), which directly computes the squared differences between image intensities, ECC focuses on the normalized values to account for intensity variations and achieve robust image alignment. To date, only a few studies have reported the application of multi-modal image registration in plant science. The systematic investigation of the performance of the registration method also focuses on the use of licensed MATLAB software [11, 15–17, 24, 25]. This project aimed to investigate automated image registration algorithms (Table 1) for pixel-perfect data registration of multi-domain image data of plants via open-source and license-free python packages.

**Table 1:** Image registration algorithms used in this study

Method	Feature	Python library	References
Phase correlation	Frequency domain	Imregpoc	Ri & Fujimoto (2018) [26]
Phase correlation + ECC	Frequency domain	Imregpoc	Ri & Fujimoto (2018) [26] Evangelidis & Psarakis (2008) [23]
ORB	Key point (Spatial domain)	OpenCV	Rublee et al. (2011) [27]
ORB + ECC	Key point & intensity (Spatial domain)	OpenCV	Rublee et al. (2011) [27] Evangelidis & Psarakis (2008) [23]
ORB Para.tuned	Key point (Spatial domain)	OpenCV	Rublee et al. (2011) [27]
ORB Para.tuned + ECC	Key point & intensity (Spatial domain)	OpenCV	Rublee et al. (2011) [27] Evangelidis & Psarakis (2008) [23]
NCC- adaptive approach	Depending of selected method	—	In this study

*Note*: Detailed parameter description of the methods in Supplementary information SI.1

Here, data from (i) RGB imaging as a basic reference method for human inspection and assessment of plant stress, (ii) hyperspectral imaging as high-dimensional data, providing biochemical information mainly on the composition of plant pigments, and (iii) chlorophyll fluorescence imaging providing high-contrast data and functional information on photosynthesis, are fused at the data/pixel level. The main objectives of this study are as follows:

1. To investigate the impact on image registration performance when the reference image of the multidomain approach used is varied.
2. To evaluate the performance of commonly used automated image registration algorithms such as feature-based ORB and phase-only-correlation of the Fourier transform are tested. Additionally, we propose a new NCC-based approach for image registration.
3. To study the effect of changing the frame/wavelength for the moving and reference images on the performance of image registration.To study the effect of changing the frame/wavelength for the moving and reference images on the performance of image registration.
4. To establish a data processing pipeline for the investigation of synergistic information via multi-domain imaging of plants via high-throughput phenotyping systems.

## Results

**Fig. 1:**
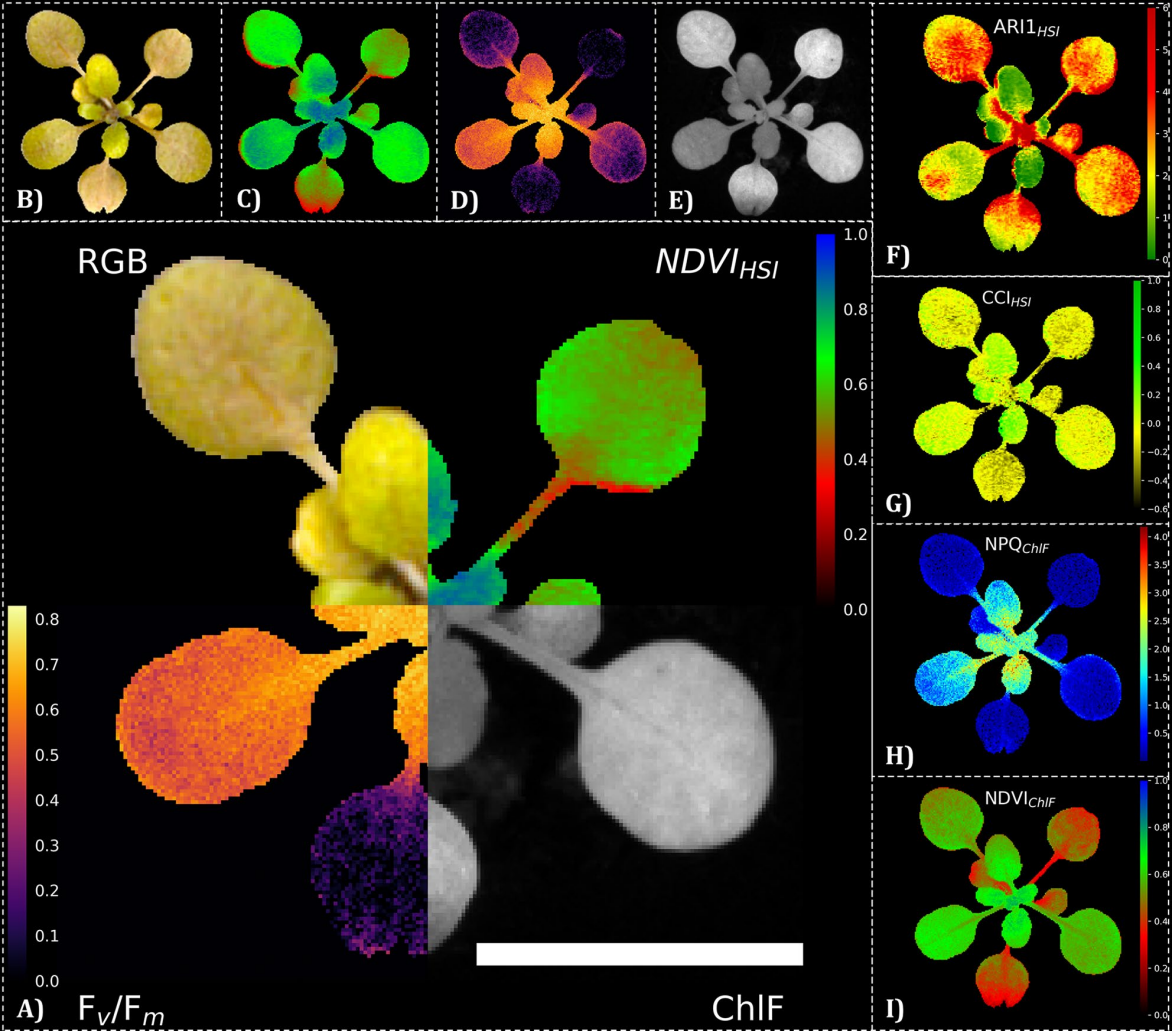
Pixel-perfect multidomain image registration of *A. thaliana*. **A**) The central image displays a montage of the registered output data of three different sensor systems: an RGB sensor, a hyperspectral imaging sensor (HSI) and a chlorophyll fluorescence camera (ChlF). **B**-**E**) Frist row shows the segmented RGB, NDVI_HSI_, and F_v_/F_m_ data and the grayscale of ChlF intensity. The images in the side column display data analysis by vegetation indices such as **F**) the anthocyanin reflectance index (ARI1), **G**) the chlorophyll: carotinoid index (CCI), **H**) the normalized difference vegetation index (NDVI) or calculated parameters from ChlF, such as **I**) non-photochemica quenching (NPQ). Images of *A. thaliana* of a salt treated variant (50 mM NaCl) and cultivated in the modified PhenoWell^®^ culture system were taken at 21 days after treatment (DAT). Image registration was performed via NCC-adaptive approach in which a transformation matrix was derived by transforming the green channel of the RGB camera and the mean intensity from 540–560 nm of the HSI camera to the far-red reflectance (730 nm) of the ChlF image sensor as the target image. The white scale bar indicates 10 mm

In this study, we demonstrate multi-modal image registration of RGB, hyperspectral (HSI) and chlorophyll fluorescence (ChlF) kinetics data (Fig. 1) and investigate the effects of target image data selection, different registration algorithms and frame selection choices on image registration performance.

### Experimental setup for multi-modal image registration

We set up a data acquisition pipeline (SI. 2) to transform image data from a sensor system (Fig. 1, HAIP BlackBox V2) consisting of an HSI system (operating between the VIS and the NIR region, 500–1000 nm) with push broom line scanner and a slightly tilted RGB camera to a chlorophyll fluorescence imager (PhenoVation Plant Explorer XS) capable of capturing various fluorescence parameters as well as red and far-red reflectance images.

The main challenges with this approach are the management of multi-modal data and the high degrees of freedom required for accurate image registration. This includes addressing for translation, rotation, scaling, and shearing, as well as accounting for potential non-linear effects (Table 2). Even if the position of the Multi-well plates under the ChlF imager was kept constant, the plates were only roughly aligned with the same orientation under the RGB and HSI sensor system. Furthermore, pixel-accurate registration of multiple objects within an image with a single global transformation matrix is exceptionally problematic, as the matrix must be estimated correctly across all subregions of the whole image.

**Table 2:** Multi-modal imaging sensor setup

Sensor	Acquisition type	Image size [px]	Scaling [% ]	Dimensionality	Calibration error [px]
RGB	Top view (slightly tilted)	1410 × 1410	132%	3 (blue, green, red)	0.44 ± 0.25 (0.31 ± 0.18)^1^
HSI	Top view (push broom)	1080 × 1080	132%	250 (2 nm sampling)	2.15 ± 1.12 (2.07 ± 1.04)^1^
ChlF	Top view	2240 × 2240	100%	13 (raw parameters)	0.26 ± 0.12 (0.11 ± 0.05)^1^

^1^*Note*: Normalized to the amount of sensor pixel. Normalized error = Calibration error x 1000/√Image size according to Stumpe et al. 2024. Camera calibration was conducted with the following frames RGB: Composite gray image, HSI: Far red reflection (728–768 nm), ChlF: Far red reflection (730 nm)

We restricted our image registration task to affine transformation because of benefits in terms of computational speed, reversibility, putative higher robustness (fewer parameters to be estimated) and minimal alteration of the original data. To address anticipated non-linear effects in the image data from each sensor — arising from factors such as imperfect optical path alignment, lens distortion, or potential geometric distortion in HSI push broom scanners due to misalignment of captured image lines — we implemented camera calibration to rectify these distortions. After camera calibration we reported the mean reprojection error (Table 2) in subpixel range, with normalized errors of 0.31 ± 0.18 and 0.26 ± 0.12 for the RGB camera and ChlF imager respectively. In the case of the hyperspectral camera, we could document a slightly higher normalized mean error of 2.07 ± 1.04 for the 25 calibration images. This higher value can be explained by a lower signal-to-noise ratio, imperfect focusing, an intensity average across multiple frames, and the inherent characteristics of line-scanner data acquisition technology.

### Effect of reference image selection on registration performance

The first step of our RGB/HSI/ChlF multi-modal image registration approach is to decide which of the sensor systems is most suitable in terms of registration performance to serve as a reference/target image. Here, we assume that we only have to estimate the transformation matrix for a single frame of each sensor system and then can apply this transformation matrix to the whole image stack of the respective sensor. However, this is not necessarily the case with HSI data due to potential chromatic aberrations.

We used a manually annotated test set (15 image trios) of *A. thaliana* subjected to different stress treatments to assess the performance of the different registration approaches for the non-stressed and stressed phenotypes (Table 3).

**Table 3:** Dataset for image registration

Plant species	Explant type	Treatments within dataset	Total image trios [RGB + HSI + ChlF]	Time points [DAT]	Test image trios [RGB + HSI + ChlF]
*A. thaliana*	Seedling	Control (WHC = 80%) Water stress (WHC = 100%) Drought stress (WHC = 50%) Salt stress (50 mM NaCl) Copper stress (200 µM CuSO4)	140	0, 3, 7, 14, 15, 21	15
*Rosa spp.*	Detached leaf disc	Control (H_2_O) D. rosae inoculated	69	0, 2, 4, 6, 8	15

**Fig. 2:**
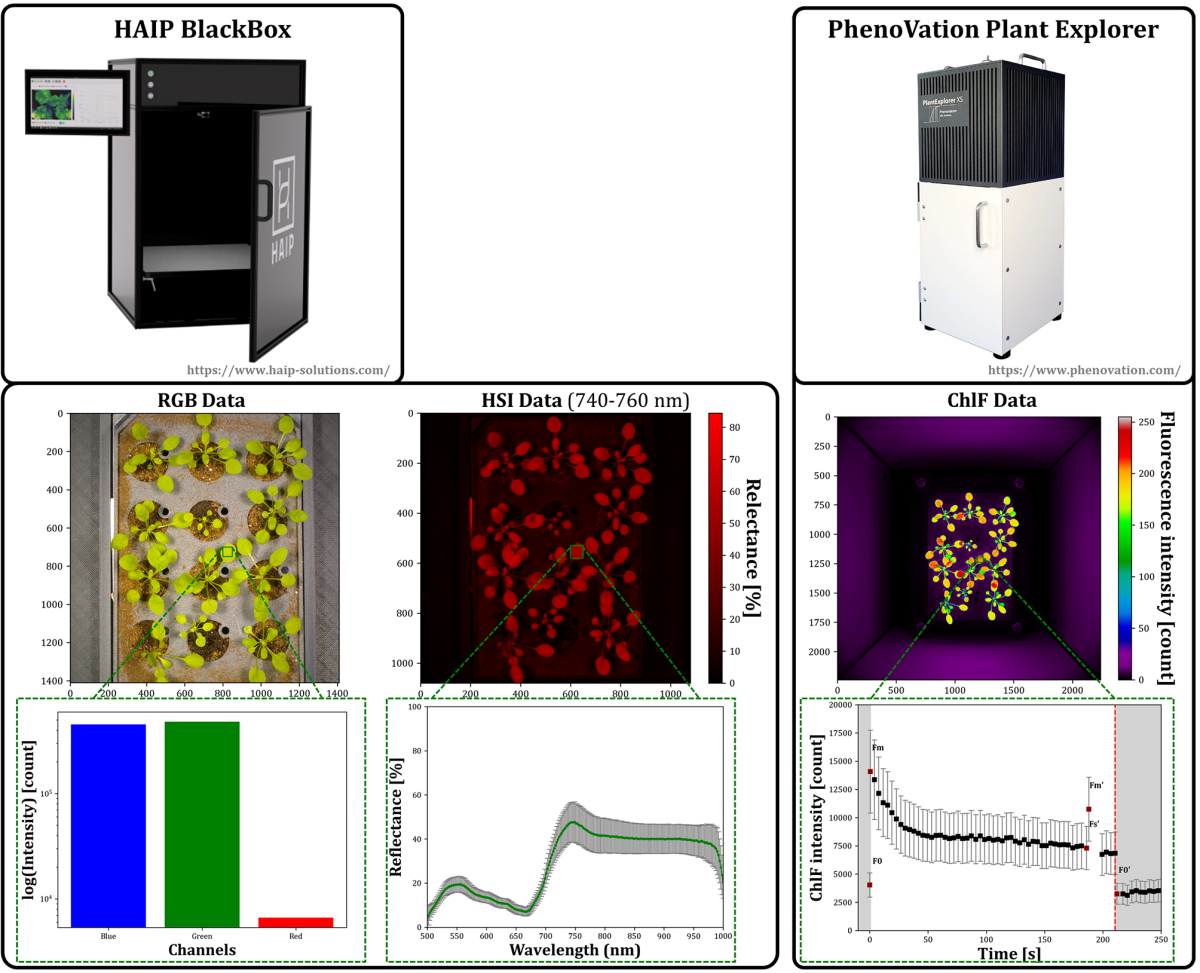
Hardware and output data of Multi-modal imaging of *A. thaliana* cultivated in modified PhenoWell^®^-plates. The upper row shows the two commercially available sensor systems (hyperspectral and chlorophyll fluorescence imager). The middle row displays exemplary output data of the used imaging sensor. The lower row shows the difference in the dimionsality of the output data of a subregion (green squares) ranging from 3 channels of RGB data, 250 spectral channels of hyperspectral camera and at least five raw/base fluorescence signals of the chlorophyll fluorescence imager (F_0_,F_m_,F_m’_,F_s’_,F_0’_ colored red in chlorophyll fluorescence kinetic data)

We applied a phase-only-correlation algorithm and investigated the registration performance (Fig. 2) via normalized cross-correlation (NCC) and the overlap ratio of convex-hull transform binary objects (OR_Convex_ or OR if related to non-convex transformed objects; see the Methods section for a detailed description). As we detected the highest NCC (and low standard deviation indicating no occurrence of total failures) with values of 0.84 ± 0.03 for RGB-to-ChlF and 0.92 ± 0.01 for HSI-to-ChlF and the highest values of OR_Convex_ of 96.6 ± 1.5% and 90.9 ± 4.1%, respectively for the transformation to ChlF data, we selected the ChlF data as the reference for further investigations. A low standard deviation indicates that no total failures have occurred. Accordingly, the ChlF data was selected as the reference for further investigations.

### Effects of the registration method on registration performance

After defining the reference image data, we investigated various registration algorithms with respect to their image registration performance (Tables 4 and 5). Here, the feature-based method with a limited number of features (ORB and ORB + ECC, max. Features = 1000) required the shortest computation time to determine the transformation matrix, but concurrently produced the highest rates of total failure (TF rate) of 31.0% and 29.0%, respectively, when transforming RGB-to-ChlF in the full dataset. As the study of Henke et al. (2019b) demonstrated that parameterization of feature-based methods is crucial for successful image registration, we included two methods with increased maximal features (ORB Para. tuned, ORB Para. tuned + ECC) [16]. None of the algorithms examined in the case of HSI-to-ChlF showed a total failure of image registration. Furthermore, POC + ECC and ORB Para.tuned + ECC for RGB-to-ChlF and ORB + ECC for HSI-to-ChlF reached the highest OR and OR_Convex_ on the test set. Upon closer inspection of the image registration performance, we observe that the algorithm achieving the highest Overlap Ratio (OR) varies across the image files and is thus file-specific. Notably, this algorithm also attains the highest normalized cross-correlation (NCC) value simultaneously. Methods based on phase correlation are robust to noise but sensitive to structurally similar patterns and nonlinear effects such as deformations [15]. Additionally, feature-based methods have the disadvantage of depending on the detection of corresponding features. Therefore, a file-specific decision could increase robustness. We therefore tested the NCC-adaptive approach, in which the method with the highest NCC value for a single file is selected. This means that the method that resulted in the highest similarity between the registered frames was selected. Even though the registration time was approximately doubled compared with that of the other methods, we were still able to further improve the image registration performance for OR_Convex_ with 98.8 ± 1.6% for RGB-ChlF (Table 4) and 94.3 ± 2.8% for HSI-ChlF (Table 5) on the test data. Moreover, the lowest standard deviation indicates a high level of robustness and the avoidance of total failure. In addition to the overlap ratio (OR, OR_Convex_), we attempt to track the registration performance on the test set by the average deviation of the centroid/midpoint (ΔMP, ΔMP_Convex_) coordinates of the manually segmented image data (see Methods section for a detailed description).

**Table 4:** Registration performance of algorithms transforming RGB-to-ChlF on the *A. thaliana* dataset

Method	Full set			Test set				
	Time [s]	TF rate [%]	NCC [-]	NCC [-]	ΔMP [px]	OR [%]	ΔMP_Convex_ [px]	OR_Convex_ [%]
POC	1.36 ± 0.10	**0.0**	0.82 ± 0.04	0.84 ± 0.03	4.90 ± 2.65	91.2 ± 2.7	2.36 ± 1.51	96.6 ± 1.5
POC + ECC	2.11 ± 0.05	**0.0**	0.82 ± 0.04	0.84 ± 0.03	5.01 ± 2.27	91.8 ± 3.1	2.29 ± 1.62	96.7 ± 1.7
ORB	**0.07 ± 0.01**	31.0	0.74 ± 0.20	0.84 ± 0.03	**4.75 ± 2.52**	91.2 ± 7.4	2.25 ± 1.80	96.5 ± 3.2
ORB + ECC	0.26 ± 0.03	29.0	0.75 ± 0.19	0.84 ± 0.03	4.87 ± 2.31	90.9 ± 5.3	2.25 ± 1.80	96.3 ± 2.3
ORB Para.tuned	17.43 ± 0.65	**0.0**	0.82 ± 0.04	0.84 ± 0.03	4.99 ± 2.58	90.2 ± 3.0	**2.25 ± 1.42**	96.1 ± 1.7
ORB Para.tuned + ECC	17.60 ± 0.65	**0.0**	0.82 ± 0.04	0.84 ± 0.03	4.85 ± 2.19	91.7 ± 3.3	2.29 ± 0.43	96.7 ± 1.7
NCC-adaptive approach	40.13 ± 1.57	**0.0**	**0.82 ± 0.04**	**0.84 ± 0.03**	4.91 ± 2.25	**92.0 ± 3.1**	2.35 ± 1.51	**96.8 ± 1.6**

*Note*: Image metrics were investigated with the following frames: RGB: Composite gray image, HSI: Far red reflection (mean of 740–760 nm), ChlF: Far red reflection (730 nm). Mean ± SD

**Table 5:** Registration performance of algorithms transforming HSI-to-ChlF on the *A. thaliana* dataset

Method	Full set			Test set				
	Time [s]	TF rate [%]	NCC [-]	NCC [-]	ΔMP [px]	OR [%]	ΔMP_Convex_ [px]	OR_Convex_ [%]
POC	0.54 ± 0.04	**0.0**	0.93 ± 0.01	0.92 ± 0.01	7.29 ± 3.68	83.0 ± 5.5	5.74 ± 2.67	90.9 ± 4.1
POC + ECC	0.88 ± 0.03	**0.0**	0.94 ± 0.01	0.94 ± 0.01	**7.16 ± 3.65**	86.8 ± 4.8	**5.21 ± 2.39**	93.0 ± 3.2
ORB	**0.04 ± 0.01**	**0.0**	0.94 ± 0.01	0.93 ± 0.01	7.46 ± 3.21	87.0 ± 4.8	5.58 ± 2.21	93.3 ± 3.4
ORB + ECC	0.15 ± 0.02	**0.0**	0.94 ± 0.01	0.94 ± 0.01	7.52 ± 3.52	**88.8 ± 4.4**	5.53 ± 2.38	**94.3 ± 2.8**
ORB Para. tuned	12.21 ± 1.46	**0.0**	0.93 ± 0.02	0.92 ± 0.01	7.48 ± 3.66	80.1 ± 3.6	5.53 ± 2.51	89.4 ± 3.2
ORB Para.tuned + ECC	12.29 ± 1.46	**0.0**	0.94 ± 0.01	0.93 ± 0.01	7.26 ± 3.78	85.4 ± 3.9	5.43 ± 2.24	92.3 ± 2.8
NCC-adaptive approach	26.71 ± 2.94	**0.0**	**0.94 ± 0.01**	**0.94 ± 0.01**	7.52 ± 3.52	**88.8 ± 4.4**	5.44 ± 2.30	**94.3 ± 2.8**

*Note*: Image metrics were investigated with the following frames: RGB: Composite gray image, HSI: Far red reflection (mean of 740–760 nm), ChlF: Far red reflection (730 nm). Mean ± SD

### Effects of frame selection on registration performance

We investigated the effect of frame selection on image registration performance. As expected, the highest NCC (0.91 ± 0.03) was detected for the Red channel and the composite gray of RGB against Red reflection of ChlF for RGB-to-ChlF (Table 6) registration. A similar pattern was observed in the HSI-ChlF registration, where the Far red and NIR reflectance of the HSI reached the highest NCC (0.94 ± 0.01) when transformed to the Far red reflectance image of ChlF (Table 7). However, the best image performance in terms of OR_Convex_ could be detected in both cases for Green channel of RGB with 97.6 ± 1.6% and Green reflection of HSI with 95.7 ± 1.9% against the chlorophyll fluorescence emission of ChlF, despite rather low NCCs of 0.31 ± 0.07 and 0.41 ± 0.12, respectively (Tables 6 and 7). This scenario reflects a true multi-domain registration as it attempts to match RGB data to ChlF data. However, testing this on the full set revealed TF rates of 10% and 13.6% for RGB-to-ChlF and HSI-to-ChlF, respectively (data not shown).

**Table 6:** Frame selection effect to image registration of RGB to ChlF on test data of *A. thaliana*

Moving/ Traget image	Metric	Blue Channel (~ 440 nm)	Green Channel (~ 550 nm)	Red Channel (~ 700 nm)	Composite gray image
Red reflection (660 nm)	NCC [-]	0.87 ± 0.02	0.90 ± 0.03	**0.91 ± 0.03**	**0.91 ± 0.03**
	ΔMP [px]	4.85 ± 2.57	4.89 ± 2.51	4.83 ± 2.57	4.96 ± 2.48
	OR [%]	89.5 ± 3.1	89.9 ± 3.2	88.9 ± 3.5	89.5 ± 3.4
	ΔMP_Convex_ [px]	2.46 ± 1.54	**2.05 ± 1.65**	2.06 ± 1.71	2.23 ± 1.59
	OR_Convex_ [%]	95.8 ± 1.8	96.0 ± 1.8	95.6 ± 2.0	95.8 ± 1.9
Far red reflection (730 nm)	NCC [-]	0.71 ± 0.07	0.86 ± 0.02	0.85 ± 0.03	0.84 ± 0.03
	ΔMP [px]	9.99 ± 14.59	**4.81 ± 2.17**	4.91 ± 2.23	4.91 ± 2.25
	OR [%]	81.6 ± 21.5	92.6 ± 2.9	91.4 ± 3.5	92.0 ± 3.1
	ΔMP_Convex_ [px]	7.55 ± 14.98	2.26 ± 1.57	2.22 ± 1.54	2.35 ± 1.51
	OR_Convex_ [%]	88.8 ± 18.5	97.1 ± 1.6	96.6 ± 1.8	96.9 ± 1.6
Chlorophyll fluorescence (> 650 nm)	NCC [-]	0.21 ± 0.05	0.31 ± 0.07	0.26 ± 0.07	0.27 ± 0.07
	ΔMP [px]	107.25 ± 69.39	4.78 ± 2.44	47.33 ± 64.60	84.83 ± 123.26
	OR [%]	7.4 ± 4.4	**93.6 ± 2.8**	58.9 ± 44.4	58.2 ± 45.5
	ΔMP_Convex_ [px]	108.57 ± 67.06	2.38 ± 1.65	45.53 ± 63.45	84.27 ± 126.42
	OR_Convex_ [%]	12.0 ± 8.2	**97.6 ± 1.6**	62.5 ± 44.6	61.4 ± 46.5

*Note*: Image metrics were investigated with NCC-adaptive approach and ChlF as reference registration target. Mean ± SD

**Table 7:** Frame selection effect to image registration of HSI to ChlF on test data of *A. thaliana*

Moving/ Traget image	Metric	Green reflection (540–560 nm)	Red reflection (640–660 nm)	Far red reflection (740–760 nm)	NIR Channel (840–860 nm)
Red reflection (660 nm)	NCC [-]	0.90 ± 0.01	0.93 ± 0.01	0.81 ± 0.06	0.81 ± 0.06
	ΔMP [px]	7.87 ± 4.35	7.91 ± 3.86	16.97 ± 37.00	7.77 ± 4.21
	OR [%]	84.8 ± 4.3	84.7 ± 4.3	78.5 ± 22.2	79.3 ± 0.22
	ΔMP_Convex_ [px]	5.51 ± 3.32	5.64 ± 2.64	15.10 ± 37.41	5.59 ± 2.86
	OR_Convex_ [%]	92.2 ± 2.7	92.0 ± 2.9	85.6 ± 23.8	85.9 ± 24.0
Far red reflection (730 nm)	NCC [-]	0.90 ± 0.01	0.83 ± 0.05	**0.94 ± 0.01**	**0.94 ± 0.01**
	ΔMP [px]	7.79 ± 4.42	7.81 ± 3.84	7.52 ± 3.52	7.31 ± 3.97
	OR [%]	89.4 ± 4.2	85.9 ± 5.1	88.8 ± 4.4	88.6 ± 4.1
	ΔMP_Convex_ [px]	5.39 ± 3.20	5.60 ± 2.92	5.44 ± 2.30	5.44 ± 2.95
	OR_Convex_ [%]	94.9 ± 2.5	92.7 ± 3.1	94.3 ± 2.8	94.2 ± 2.6
Chlorophyll fluorescence (> 650 nm)	NCC [-]	0.41 ± 0.12	0.25 ± 0.07	0.62 ± 0.15	0.55 ± 0.15
	ΔMP [px]	7.82 ± 4.40	126.14 ± 42.28	7.37 ± 4.08	**7.13 ± 4.10**
	OR [%]	**91.0 ± 3.4**	4.4 ± 6.4	90.1 ± 3.4	88.5 ± 9.1
	ΔMP_Convex_ [px]	**5.39 ± 3.11**	126.31 ± 42.54	5.52 ± 2.80	5.03 ± 2.71
	OR_Convex_ [%]	**95.7 ± 1.9**	6.2 ± 8.7	95.2 ± 1.8	93.8 ± 6.7

*Note*: Image metrics were investigated with NCC-adaptive approach and ChlF as reference registration target. Mean ± SD

**Fig. 3:**
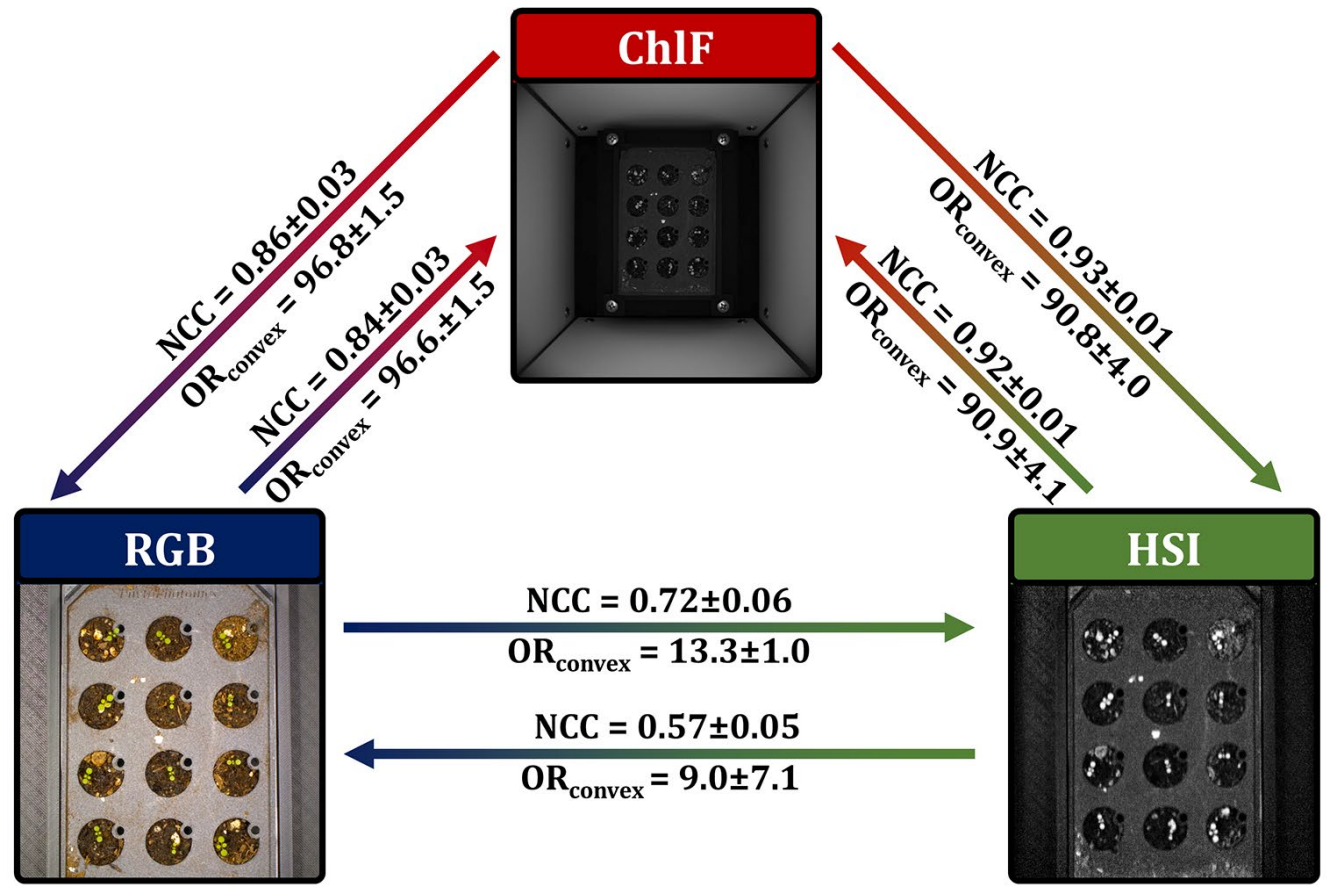
Effect of reference image selection on the registration performance on the *A. thaliana* test dataset. Image metrics were investigated with phase-only-correlation algorithms and the following frames RGB: composite gray image, HSI: Far red reflection (740–760 nm), ChlF: Far red reflection (730 nm)

To visualize the performance on the test data we created a Pseudo-RGB image consisting of the manually annotated RGB binary mask as the blue channel, the HSI binary mask as the green channel and ChlF binary mask as red channel (Fig. 3).

In this study, we observed that image registration performance was superior near the center of the image compared to regions further away from the center. For the complete dataset, registrations using the Green channel and Green reflection to Far Red reflection of ChlF demonstrated a TF rate of 0.0% in both cases. The normalized cross-correlation (NCC) was 0.84 ± 0.03 for RGB-to-ChlF and 0.87 ± 0.03 for HSI-to-ChlF.

**Fig. 4:**
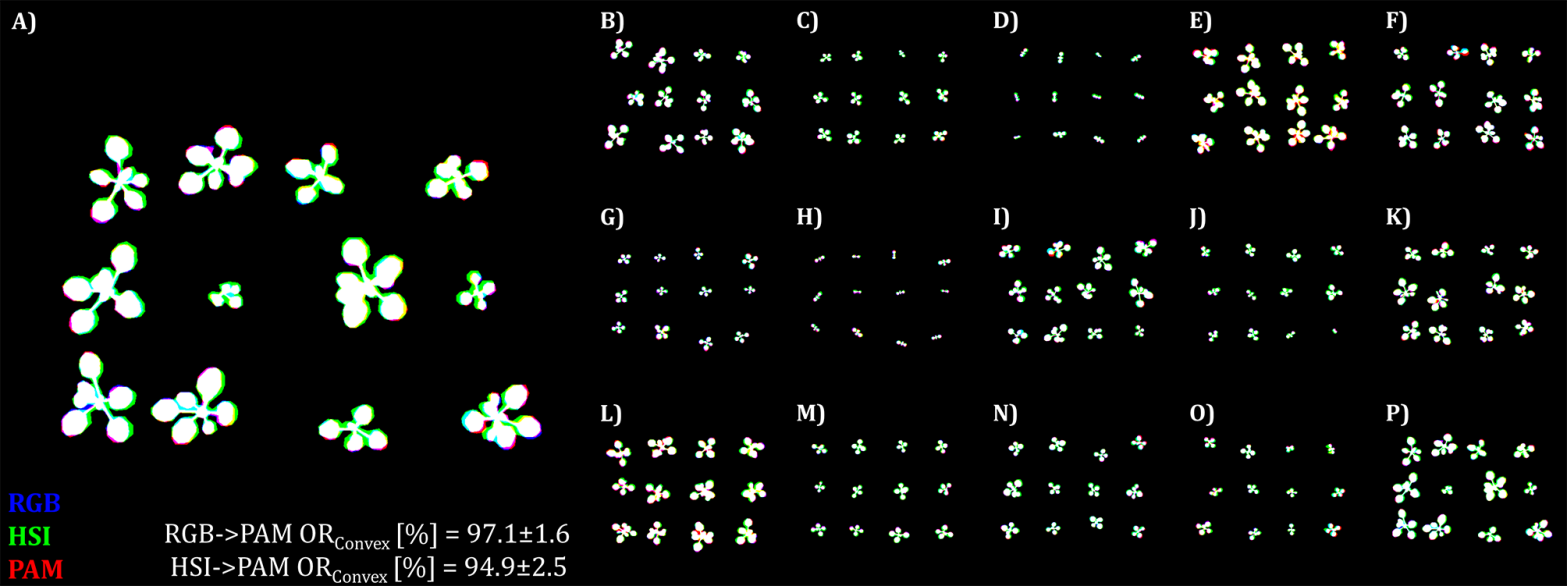
Illustration of the image registration performance of the NCC-adapative approach on test sets of *A. thaliana* via visualisation of the intersection. **A**) shows an enlarged version of one of the images from the test set (**B**–**P**). Pseudo RGB was composed of manually annotated binary images of test image trios. Here, the blue color represents the binary mask of the RGB dataset, the green color represents the binary mask of the HSI and the red color for the binary mask of the ChlF imaging sensor. Intersecting pixel of the two performed registrations (RGB→ChlF, HSI→ChlF) are coloured white, while violett represents the intersection between the RGB and ChlF data, and cyan represents the intersection between the RGB and the HSI data and yellow the HSI and the ChlF data overlap. Image registration was conducted with the NCC-adaptive approach and the following frames RGB: Green channel, HSI: Green reflection (540–560 nm), ChlF: Far red reflection (730 nm)

Although the image data were only captured at a single time point, the fixed position of the Multi-well plate in the chlorophyll fluorescence camera allowed for monitoring changes over time. Figure 4 illustrates the segmented image data, highlighting changes in chlorophyll fluorescence (ChlF) for a single *A. thaliana* plant over time. Minor deviations at the leaf edges were observed only at later time points (greater than 7 days after treatment, DAT). These deviations could be attributed to slight leaf movements during data acquisition (approximately 5 min per sample) or sample transportation between sensor systems. Alternatively, they may result from inadequate scaling of the hyperspectral imaging (HSI) data by the estimated transformation matrix, since key point detection may be limited by the low contrast characteristics of the data. The manifestation of stress symptoms at 21 DAT in the shown plant of a control variant, indicates either limitations of the cultivation time in the multi-well plate system or the effect of an uncontrolled parameter in the new cultivation system, such as heat stress or nutrient deficiency. But here only serves to demonstrate the registered image data.

### Application to detached leaf assay of *Rosa × Hybrida*

Once various factors influencing image registration performance were investigated and optimized for *A. thaliana*, we applied the NCC-adaptive approach to multi-modal image data from a detached leaf assay of the black spot disease highly susceptible rose cultivar ‘Pariser Charme’ [27–29]. In the case of *A. thaliana*, the best image registration performance was reached when the transformation matrix from Green channel of RGB and Green reflection channel of HSI to Chlorophyll fluorescence frame of ChlF was estimated; however, this failed entirely for the leaf disc image data (Tables 8 and 9). Instead image registration of Green channel and Green reflection to Far red reflection of ChlF resulted in highly accurate image registration with nearly subpixel-accuracy for RGB-to-ChlF, as indicated by a low deviation of the mean midpoint ΔMP = 1.41 ± 1.00 and OR_Convex_ = 98.4 ± 0.3. HSI-to-ChlF resulted in a slightly lower performance of ΔMP = 2.09 ± 1.20 and OR_Convex_ = 96.8 ± 0.4. On the full dataset, both registrations resulted in 0.0% TF rate and NCC of 0.83 ± 0.01 for RGB-to-ChlF and 0.90 ± 0.01 for HSI-to-ChlF.

**Table 8:** Frame selection effect to image registration of RGB to ChlF on test data of *Rosa spp*

Moving/ Traget image	Metric	Green Channel (~ 550 nm)	
Far red reflection (730 nm)	NCC [-]	**0.83 ± 0.01**	
	ΔMP [px]	**1.41 ± 1.00**	
	OR [%]	**98.3 ± 0.3**	
	ΔMP_Convex_ [px]	**1.43 ± 0.97**	
	OR_Convex_ [%]	**98.4 ± 0.3**	
Chlorophyll fluorescence (> 650 nm)	NCC [-]	0.43 ± 0.02	
	ΔMP [px]	104.24 ± 72.24	
	OR [%]	29.9 ± 7.4	
	ΔMP_Convex_ [px]	104.06 ± 71.95	
	OR_Convex_ [%]	30.2 ± 7.4	

*Note*: Image metrics were investigated with NCC-adaptive approach and ChlF as reference registration target. Mean ± SD

**Table 9:** Frame selection effect to image registration of HSI to ChlF on test data of *Rosa spp*

Moving/ Traget image	Metric	Green reflection (540–560 nm)
Far red reflection (730 nm)	NCC [-]	**0.91 ± 0.01**
	ΔMP [px]	**2.09 ± 1.20**
	OR [%]	**96.7 ± 0.4**
	ΔMP_Convex_ [px]	**2.27 ± 1.11**
	OR_Convex_ [%]	**96.8 ± 0.4**
Chlorophyll fluorescence (> 650 nm)	NCC [-]	0.67 ± 0.11
	ΔMP [px]	16.62 ± 34.32
	OR [%]	85.3 ± 24.2
	ΔMP_Convex_ [px]	16.9 ± 34.3
	OR_Convex_ [%]	85.5 ± 24.1

*Note*: Image metrics were investigated with NCC-adaptive approach and ChlF as reference registration target. Mean ± SD

**Fig. 5:**
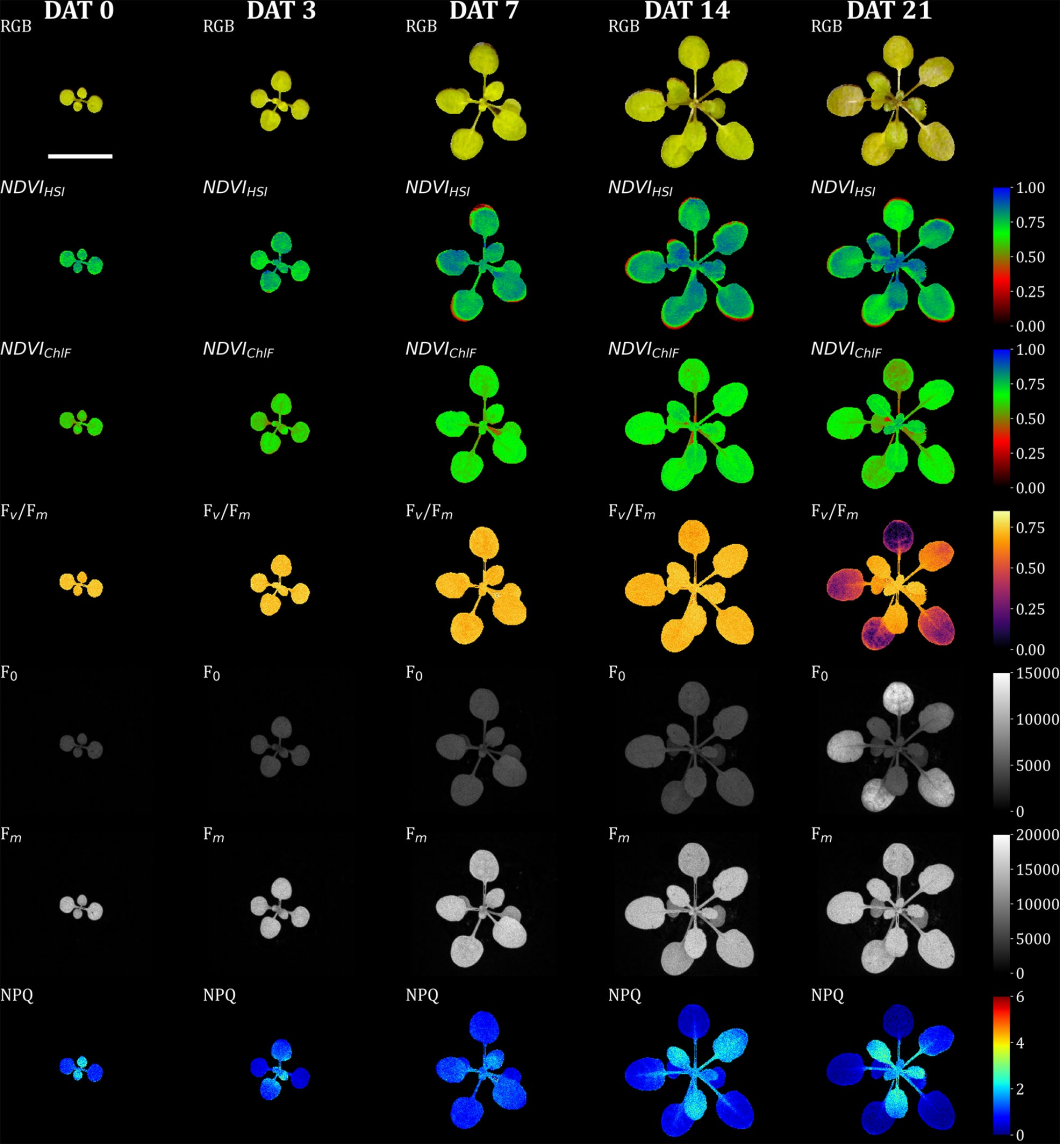
Full-registered and segmented image dataset of RGB, HSI, and ChlF data of *A. thaliana*. The Seedlings were precultivated for 7 days, after which experimental treatments were applied (defines as days after treatment 0). Here, the data of a control plant are presented over time. The frist row displays the RGB data. The second row illustrates the normalized difference vegeation index (NDVI) of HSI data. Rows 3 to 6 show different parameters (maximun quantum yield of photosynthesis: F_v_/F_m_; ground fluorescence: F_0_; maximum fluorescence: F_m_; non-photochemical quenching: NPQ) from ChlF imaging. Image registration was performed via NCC-adaptive approach in which transformation matrices were derived by transforming the green channel of the RGB camera and the mean intensity from 540–560 nm of the HSI camera to the far-red reflectance (730 nm) of the ChlF image sensor as the target image. Each image stack (DAT 0, 2, 4, 6, 8) was registered individually. Scale bar = 10 mm

We observed a similar improvement in image registration performance near the image center, consistent with our findings in *A. thaliana* (Fig. 5). It seems to be more obvious for the registration of HSI-to-ChlF (green-to-red) than for RGB-to-ChlF (blue-to-red).

**Fig. 6:**
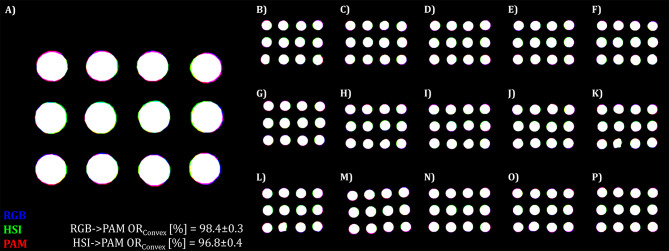
Illustration of the image registration performance of the NCC-adapative approach on the test set of *Rosa × hybrida* via visualisation of the intersection. **A**) shows an enlarged version of one of the images from the test set (**B**–**P**). Pseudo RGB was composed of manually annotated binary images of test image trios. Here, the blue color represents the binary mask of the RGB dataset, the green color represents the binary mask of the HSI and the red color for the binary mask of the ChlF imaging sensor. Intersecting pixel of the two performed registrations (RGB→ChlF, HSI→ChlF) are coloured white, while violett represents the intersection between the RGB and ChlF data, and cyan represents the intersection between the RGB and the HSI data and yellow the HSI and the ChlF data overlap. Image registration was conducted with the NCC-adaptive approach and the following frames RGB: Green channel, HSI: Green reflection (540–560 nm), ChlF: Far red reflection (730 nm)

With successful image registration, we were able to monitor the inoculation of *D. rosae* in the detached leaf assay of *Rosa × hybrida* in a multi-domain manner (Fig. 6). These preliminary results highlighted the early response (DPI 4) of the ChlF parameters to inoculation. With respect to image registration performance, no misalignment became obvious in the exemplary data (Fig. 6).

## Discussion

**Fig. 7:**
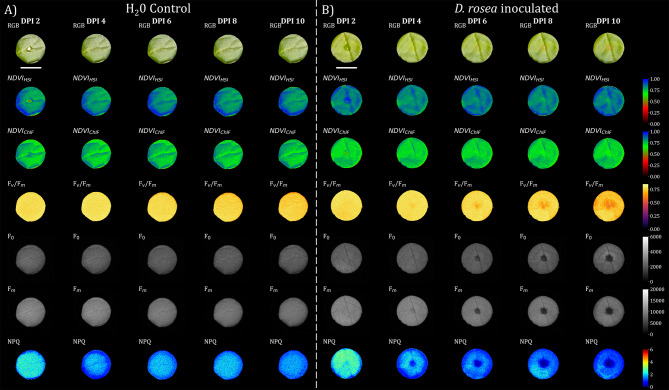
Full-registered and segmented image dataset of RGB, HSI, and ChlF data from detached leaf assay of *Rosa × hybrida.* Leaf discs were inoculated with either **A**) H_2_O or **B**) *Diplocarpon rosae* spore suspension (in this case with a concentration of 200,000 conidia/mL) for 3 days (DPI 0 - DPI 2), and the suspension was removed at DPI 2 (days post inoculation). The frist row displays the RGB data where the control/inoculum droplet is still visible at 2 DPI. The second row illustrates the NDVI from the HSI data. Row three also shows the NDVI however derived from the ChlF sensor, which was calculated based on reflection of wavelength 660 nm and 730 nm. Rows 4 to 7 show different parameters (maximun quantum yield of photosynthesis: F_v_/F_m_; ground fluorescence: F_0_; maximum fluorescence: F_m_; non-photochemical quenching: NPQ) from chlorophyll fluorescence imaging. Image registration was performed via NCC-adaptive approach in which transformation matrices were derived by transforming the green channel of the RGB camera and the mean intensity from540-560 nm of the HSI camera to the far-red reflectance (730 nm) of the ChlF image sensor as the target image. Each image stack (DAT 0, 2, 4, 6, 8) was registered individually. Scale bar = 10 mm

In this study, we systematically investigated different effects on the performance of multi-domain image registration. As the utilized ChlF camera also provided reflection-based frames (Red reflection, Far red reflection), this study was not restricted to the registration of fluorescence images from this sensor. The described multi-domain approach successfully registers data from different sensor systems, which represent deviations within the images such as tilted imaging angles, deviating wavelengths in exposure and/or detection or detector properties. The potential factors for optimizing image registration performance appear to be vast. This study examines a specific subset of these factors, while excluding certain promising methods, such as image preprocessing techniques and mutual information strategies. Previous studies on multi-modal image registration in plants have focused on the effects of image scaling for phase correlation [15] and preprocessing for feature-based registration [16]. In our study we concentrated on the selection of the reference sensor system, the method and the frame selection of the RGB, HSI and ChlF sensor systems used. In addition to the countless ways of performing image registration, the complexity of quantifying registration success—particularly with multi-modal image registration—turns the pursuit of pixel-perfect (Fig. 7) and automated registration into an extremely challenging task. Since there is no universal metric that can be optimized to guarantee the success of multi-modal image registration, manual labeling or segmentation of test images is one way to quantify registration accuracy. However, this approach is also influenced by the data quality-driven accuracy of labeling (projected plant area by HSI approx. 120%, and by RGB approx. 101% of the projected plant area by ChlF). Thus, it can be affected by variations in the scene representation and the different resolutions of the output data across different sensor systems. We therefore developed two strategies to address the problem of varying data quality for labeling in the test set. First, we calculate OR and ΔMP additionally on convex-hull-transformed binary objects (OR_Convex_, ΔMP_Convex_) to minimize degradation due to unlabeled tiny plant structures. Second, the OR is related to the smallest represented plant area of the two sensor systems, since the intersection can never be larger than the smallest unit of the overlapping areas.

Furthermore, we focused on normalized cross-correlation (NCC) as a similarity metric because of its robustness and invariance to intensity differences and contrast in unimodal image registration. However, in multi-modal image registration, the assumption of a linear relationship between image intensities is often not valid. Nonetheless, the inherent edges of plant structures may retain their relative correlation across different sensor systems, providing a potential basis for intensity and feature-based registration. Figure 2 revealed that both registrations (RGB-ChlF and HSI-ChlF) performed well under phase-only-correlation when ChlF was set as the reference image. In that case, despite the higher NCC, the OR_Convex_ of ~ 91% for HSI-to-ChlF was considerably lower than that of ~ 97% for RGB-to-ChlF. This observation can be at least partially attributed to lower resolution and the lower contrast of HSI data, which is also illustrated by a > sixfold greater normalized reprojection error of 2.07 ± 1.04 px for the HSI compared with 0.31 ± 0.18 px for the RGB camera calibration (Table 2).

Following this approach, we investigated various registration methods, including those operating in the frequency domain, in the spatial domain, and intensity-based methods, to estimate affine transformations. For the transformation of RGB to ChlF (Table 4) phase-only-correlation with coupled enhanced cross correlation (POC + ECC) outperformed the other tested algorithms in terms of robustness (0% TF rate), speed (2.11 ± 0.05 s per single transformation) and high accuracy (OR_Convex_ = 96.7 ± 1.7%) for the *A. thaliana* dataset. Since the algorithm that achieves the highest OR/OR_Convex_ value varies across all the image files and also has the highest NCC value, we included the NCC-adaptive approach. This approach could further increase the OR/ORConvex value (ORConvex = 96.8 ± 1.6%), but at the expense of speed (40.13 ± 1.57 s). The registration of RGB-to-ChlF via the NCC-adaptive approach relies on ORB.tuned + ECC in 59.3% of full set cases, ORB + ECC in 20% and on the POC + ECC method in 17.1% of the full-set cases. In case of HSI-to-ChlF (Table 5), feature-based ORB coupled with ECC seems most suitable in terms of robustness (0% TF rate), speed (0.15 ± 0.02 s) and accuracy (OR_Convex_ = 94.3 ± 2.8%). The NCC-adaptive approach could not outperform ORB + ECC in terms of test metric, as it used in 86.6% of the cases ORB + ECC (based on highest NCC), and thus both methods reached equal accuracy. On full set, NCC-adaptive approach used in 77.1% ORB + ECC, 15.7% ORB Para.tuned + ECC and 7.1% POC + ECC for the tested registration HSI-to-ChlF.

To our knowledge, this study is the first to investigate in detail the effect of frame selection on image registration performance in multi-modal registration in plant science (Tables 6 and 7). Usually either composite gray images or preprocessed edge images are used for fluorescence to RGB transformation [11, 15, 16], but the effect of varying single images has not been investigated thus far, and is becoming increasingly interesting with the increasing relevance and use of hyperspectral cameras. As expected, selecting frames of different systems with similar wavelengths of reflected imaging scenery led to the highest NCC values (Table 6; RGB: red channel (~ 700 nm) vs. ChlF: Red reflection (~ 660 nm); Table 7; HSI: Far red reflection (740–760 nm) vs. Far red reflection (~ 730 nm) and at the same time to a remarkable registration performance. Using the Green channel from RGB (Table 6) instead of the composite gray image slightly increased the performance of image registration on the test set. In addition, image registration performance using Green channel of the HSI (Table 7) is superior with respect to the OR/OR_Convex_ value to the Red/Far red or NIR reflection frame of HSI when the Far red reflection of ChlF is used as target image.

Interestingly, the highest accuracy in terms of OR and OR_Convex_ could be reached in both registrations with the use of Green reflection frame (RGB&HSI) to fluorescence image (ChlF; long integrated F_m_ of PAM imager). This effect might be associated with a varying signal(plant)-to-noise(background) ratio (SNR), which is with a decreasing SNR for RGB: G > R > B, HSI: FR > G > R, and ChlF: ChlF > > FR > > R. A detailed inspection of single files of the full set where total failure of image registration occurred (TF rate of 10% and 13.6% for RGB-to-ChlF and HSI-to-ChlF, respectively) is needed to increase robustness when estimating the transformation matrix from these frames.

To evaluate the generalizability of the registration pipeline, we applied the best-performing approaches to the detached leaf assay of *Rosa × hybrida*. Owing to the simple shape of round leaf disc, this system allows easier quantification of the registration accuracy. Indeed, estimating the transformation matrix from the green channel of the RGB (Table 8) or Green reflection of the HSI (Table 9), respectively, to Far red reflection of ChlF resulted in a high overlap ratio OR/OR_Convex_ indicating close-to-pixel-perfect registration performance. The low performance of both registrations (RGB-to-ChlF and HSI-to-ChlF) when the fluorescence image was used as the target image can be attributed in part to the inoculation-dependent spatial heterogeneity of fluorescence frame (Fig. 6).

Nevertheless, to our knowledge, this study is the first to report this drastic reduction in fluorescence after the inoculation of *Rosa* spp., with the first symptoms already observed after DPI 4 (days post inoculation). However, given that the spores were harvested from field-grown roses, it remains unclear whether the observed symptoms are caused exclusively by *D. rosae*. Conclusive identification of the spores in the suspension would necessitate genetic analysis or microscopic examination. In addition, these preliminary data need to be validated in more detail in a separate study, particularly with respect to their use as automated phenotyping tool to quantify the magnitude of tolerance/resistance mechanisms of Rosa genotypes against black spot disease.

To date, only a limited number of image registration approaches (reviewed in the preprint of Stumpe et al. 2024 [18]) applied to plant science have been reported. Most of them are restricted to fusing a single object of interest the data of unimodal or multi-modal sensor systems. This study focused instead on extending high-throughput phenotyping to take advantage of multi-modal imaging by simultaneously acquiring multiple objects at once. Pixel-accurate registration of multiple objects within an image with a single global transformation matrix is exceptionally problematic, as the matrix must be estimated correctly across all subregions of the whole image. However, we qualitatively observed that the registration performance decreases with increasing extension toward the edges of the image (Figs. 3 and 4). We attribute this to imperfect camera calibration, so that non-linear effects persist in the images with correction for lens distortion. Nevertheless, splitting the whole image into smaller regions — by the isolation of the objects on basis of their centroids — should further increase the registration performance, since non-linear relations of the whole image are now restricted to the region of interest. In this way, we could report a further increase in image registration performance of both registration (RGB-to-ChlF and HSI-to-ChlF) and datasets (Table 10: *A. thaliana*, Table 11: *Rosa × hybrida*). In the case of *A. thaliana*, this approach becomes more challenging when the cultivation of *A. thaliana* in Multi-well plates is extended to 3–4 weeks, as the individual plants begin to overlap, causing isolation to become problematic. In summary, for both registrations (RGB-to-ChlF and HSI-to-ChlF) we can report an overall OR_Convex_ of 97.3% for *A. thaliana* and 98.6% for *Rosa × hybrida* detached leaf assay with the best performing approach on test data. Thus, an additional fine registration increased the mean OR_Convex_ value for *A. thaliana* by 1.0% and for *Rosa × hybrida* by a total of 0.3%. A further increase in image registration could be expected by reducing the reprojection error for instance by deconvolution of HSI data (Zabic et al. 2024, unpublished [31]) or by applying non-rigid transformation in fine registration stage.

**Table 10:** Effect of fine registration by the second stage with single-plant images

*A. thaliana*	Sample	NCC [-]	ΔMP [px]	OR [%]	ΔMP_Convex_ [px]	OR_Convex_ [%]
**RGB-to-ChlF**						
Single stage*	15 × 12 = 180	0.86 ± 0.02	1.78 ± 1.14	93.1 ± 4.4	1.69 ± 1.14	97.4 ± 2.5
Second stage	15 × 12 = 180	0.91 ± 0.02	1.41 ± 0.86	94.4 ± 3.7	1.32 ± 0.89	98.0 ± 2.3
**HSI-to-ChlF**						
Single stage*	15 × 12 = 180	0.90 ± 0.01	3.70 ± 1.78	89.4 ± 6.6	3.64 ± 1.80	95.2 ± 4.0
Second stage	15 × 12 = 180	0.93 ± 0.02	3.38 ± 1.90	91.8 ± 6.9	3.39 ± 1.93	96.6 ± 4.2

*Note*: Image metrics were investigated with NCC-adaptive approach and ChlF as reference registration target. Mean ± SD. *As in Tables 5 and 6, but the averaging was applied to the images of single objects instead of the images at plate level with 12 individual objects

**Table 11:** Effect of fine registration by the second stage with single-plant images

*Rosa × hybrida*	Sample	NCC [-]	ΔMP [px]	OR [%]	ΔMP_Convex_ [px]	OR_Convex_ [%]
**RGB-to-ChlF**						
Single stage*	15 × 12 = 180	0.83 ± 0.01	1.33 ± 0.85	98.6 ± 0.6	1.38 ± 0.83	98.7 ± 0.6
Second stage	15 × 12 = 180	0.91 ± 0.02	1.11 ± 0.79	98.8 ± 0.5	1.22 ± 0.76	98.9 ± 0.5
**HSI-to-ChlF**						
Single stage*	15 × 12 = 180	0.91 ± 0.01	2.62 ± 1.40	98.0 ± 1.0	2.68 ± 1.45	98.0 ± 1.0
Second stage	15 × 12 = 180	0.97 ± 0.01	2.53 ± 1.50	98.3 ± 1.3	2.54 ± 1.54	98.3 ± 1.3

*Note*: Image metrics were investigated with NCC-adaptive approach and ChlF as reference registration target. Mean ± SD. *As in Tables 7 and 8, but the averaging was applied to the images of single objects instead of the images at plate level with 12 individual objects

## Conclusions

In the present study, we have demonstrated successful multi-modal image registration of RGB/HSI/ChlF data with accuracy partially up to single-pixel, where one pixel in our case represents an area of 0.01 mm². This enables the following: (i) high-throughput, high-dimensional phenotyping of *A. thaliana* with respect to various abiotic stressors if coupled with the modified version of PhenoWell^®^, (ii) systematic investigation of the intensity relationship between RGB/HSI/ChlF by correlation analysis; and (iii) studies on the performance of ML models with increased cross-sensitivity to various abiotic stressors through the fusion of the data and on the other hand research on the optical features of*D. rosae* inoculation and their use for automated phenotyping. The latter could thus provide phenotypic data for genome-wide-association studies to identify further resistance genes against black spot disease. Data-fused multi-sensor imaging has the potential to find new proxies of the plant stress state, enhanced the cross-specificity of multiple plant stress and combined plant stress detection and provide new insights into the physiological causes of specific plant‒pathogen interactions.

## Methods

### Design of the cell culture insert for a. Thaliana

**Fig. 8:**
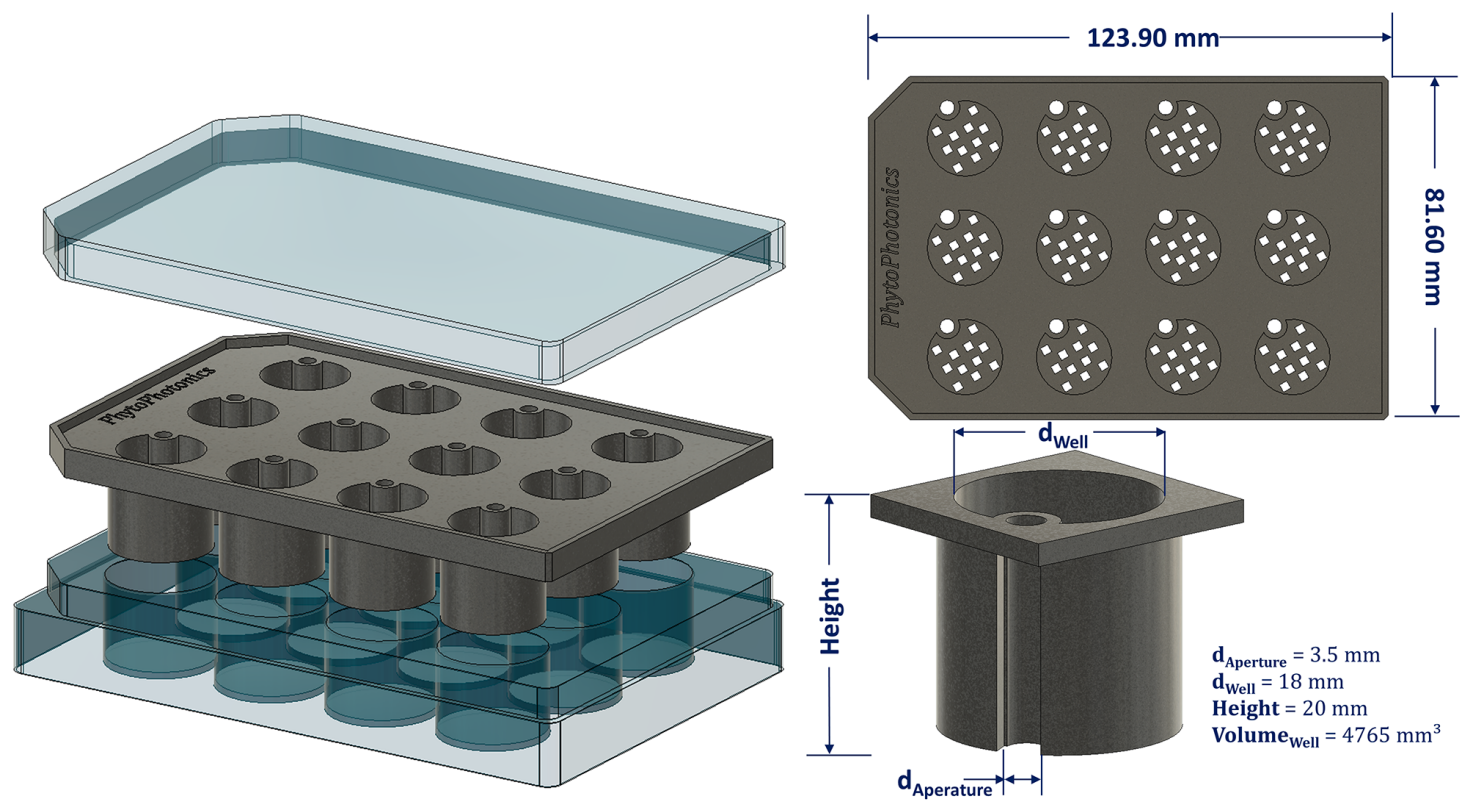
Schematic illustration of the modified inserts based on PhenoWell^®^ developed by Li et al. 2023 [5] for Multi-Well cell culture plates. The inserts were 3D-printed on commission with multi-jet fusion process from polypropylene (HP 3D HR PP, BASF, Germany)

On the basis of the recently published study of Li et al., 2023 [5], a modified version of the PhenoWell^®^ approach was designed and tested. The modified inserts (Fig. 8) for Multi-Well cell culture plate were 3D-printed on a commission with multi-jet fusion process from polypropylene (HP 3D HR PP, BASF, Germany) to provide high chemical resistance, low water absorption and autoclavability. The insert plate consisted of 12-wells, and each well provided a volume of 4.77 cm³ for the growth of *A. thaliana*. The wells were filled with a premixed substrate consisting of peat and perlite at a ratio of 9:1 (v/v). The substrate was then compressed with a 3D-printed counterpart of the inserts, which protruded 3 mm into a well, to obtain a homogeneous filling of each well. In total 35 plates were prepared for this experiment.

### Seedling growth and culture conditions of *A. thaliana*

The seeds of *Arabidopsis thaliana* Col-0 stored at 4 °C were sown in excess in each well and germinated by adding 3 ml of deionized water to the 25 inserts of the 12-well cell culture plates (Falcon^®^, 12 Well Clear Flat bottom TC-Treated; neoLab Migge GmbH, Heidelberg, Germany) filled with substrate. The lids of the cell culture plates were removed 7 days after sowing (DAS 7). The plants were cultivated at 21 °C under 100 µmol m^− 2^s^− 1^ white LED bars (GreenPower LED research module white, Philips, Netherlands) with a photoperiod of 12 h light. At 7 DAS, excessive seedlings were thinned and at 10 DAS the different stress treatments were applied (defines days after treatment, 0 DAT). The current weight of each plate was determined every 2 days and used to calculate and pipette an individual water supply depending on the specific treatment (treatments were not the focus of the study but included the following: control (WHC = 80%), water stress (WHC = 100%), drought stress (WHC = 50%), salt stress (50 mM NaCl), and copper stress (200 µM CuSO_4_)). After treatment at 0 DAT, the seedlings were cultivated for 3 weeks (DAS 31/ DAT 21) at a relative humidity of 40%.

### Detached leaf assay of Rosa × hybrida and inoculation of *D. rosae*

For the detached leaf assay, freshly unfolded rose leaves were detached, quickly surface disinfected with 50% (v/v) ethanol and rinsed with deionized water. Leaf discs of the *Rosa × hybrida* cultivar ’Pariser Charme‘ donor plants were excised via a 16 mm diameter cork drill. The culture medium consisted of 0.5% plant agar (w/v) (Duchefa, Harlem, The Netherlands) with 0.003% benzimidazole and 0.5% (w/v) active charcoal. After autoclaving 6 ml of culture media was poured into the wells of the multi-well culture plate. The fungal pathogen *D. rosae* was harvested by microspore suspension culture from field-grown rose donor plants of same cultivar and then propagated on susceptible leaves for use in this study [28]. The inoculation of the leaf discs was accomplished either with deionized H_2_O or with defined spore concentrations (50,000/100,000 or 200,000 conidia/mL suspension). A 10 µl droplet of solution was applied to each leaf disc. This droplet remained on the leaf for 2 days and was then removed with a paper towel (DPI 2). To prevent specular lighting while imaging the leaf discs a modified version of the plate insert (with the same dimensions, 3D printed with PLA) acting as a mask was used.

### Multi-modal image acquisition

Multi-modal imaging was performed at the time points DAT 0, 3, 7, 14, and 21 for *A. thaliana* and DPI 2, 4, 6, 8, and 10 for *Rosa × hybrida* and included RGB, HSI and ChlF imaging. All culture plates were dark adapted for at least 25 minutes to acquire chlorophyll fluorescence kinetic measurements with a ChlF imager (PlantExplorer XS, PhenoVation B.V., Netherlands). All images were acquired with a resolution of 2240 × 2240 px. The device specific protocol includes the acquisition of dark-adapted fluorescence parameters such as F_0_ and F_M_; light-adapted fluorescence parameter such as F_0’_,F_s’_, and F_M’_; as well as reflection-based parameter such as R_660nm_ and R_730nm_. Light adaptation was ensured by actinic light (blue LEDs with maximum emission at 450 nm) at a light intensity equal to the light intensity during cultivation of 100 µmol m^− 2^s^− 1^ for 180 s (which was proved to be sufficient to achieve the steady-state plateau in a previous study). The working distance between the sample and the camera kept constant at 270 mm.

Directly after chlorophyll fluorescence measurement, HSI and RGB images were captured with an internal scanning push broom imaging system (BlackBox V2, HAIP Solutions GmbH, Germany), equipped with a 12 mm variable focus lens (Azure). The HSI data cube was acquired with 1080 × 1080 px spatial resolution (number of total pixels) and spectral sampling of 2 nm with 250 spectral channels within the range of 500–1000 nm. The RGB images were acquired with the same device with a spatial resolution of 2688 × 1512 px and equipped with a 16 mm variable focus lens. Factory settings of the device cropped RGB data to 1410 × 1410 px to match the field of view of HSI sensor. The working distance between sample and camera was kept constant at 470 mm.

### Image registration & camera calibration & data analysis

To test different image registration methods, we first developed a python library [32] to consistently read and process the multi-modal image data. This library, which is based mainly on Numpy and OpenCV, enables the reading of multi-sensor data, processing methods such as white balancing of RGB and HSI data, as well as calculations of ChlF kinetic parameter, such as NPQ, ETR, and ΦPSII etc. To undistort the images, 25 checkerboard images (14 × 9 pattern with 5 mm squares) were acquired for each sensor systems. The intrinsic camera calibration parameters were calculated via the OpenCV implementation of Zhang’s method (2000) [33], with the calibration restricted to a single focal plane and single observer (camera) for each sensor system.

Various algorithms and images/wavelengths of the sensor systems were used for image registration. Despite static cropping of the ChlF data from 2240 × 2240 px to 1080 × 1080 px around the image center, no additional preprocessing was performed unless mentioned in the SI. 1. All the tested image registration approaches were restricted to affine transforms (SI. 2).

To visualize the syerngies of regsitered multi-domain data, several chlorophyll fluorescence parameter and vegetation indices were calculated based on formulas described in literature, exact equation can be found in SI. 3.

### Manual annotation of the projected plant area for the test set

To evaluate the image registration performance for each sensor system, 15 images from different timepoints (Table 3) were randomly selected and the projected plant area was manually labeled with the graphical user interface Roboflow^©^ [34]. Here, the annotation was conducted on the gray composite for RGB, on the frame of far red at 730 nm for HSI, and on the Far red reflection frame (~ 730 nm) for ChlF. This resulted in 15 image trios for each plant species, consisting of 12 plant replicates per image.

### Evaluation metrics of image registration performance

The registration of images from single sensor systems could theoretically achieve pixel-perfect registration (neglecting camera calibration errors) or, in other words, 100% overlap of the objects of interest in the images. However, in multi-domain image registration varying quality of the output data, e.g., low spatial resolution of HSI data, poses a challenge when evaluating image registration performance. Therefore, we developed the following similarity metrics to quantify image registration performance while minimizing the error of projected plant area annotation in the different domain sensor systems. Here, the overlap ratio of binary images A and C or B and C of the image trios (A, B, C) of the test set, describes the intersection of the output data of two imaging sensors, divided by the lowest value of the projected plant area Eq. 2, since the intersection can never be larger than the smallest subset. OR_Convex_ was introduced to account for the varying quality of labeling and the associated errors by calculating the overlap ratio in the convex representation of binary objects in the images via Eq. 3. In addition, the deviation of the center point (MP) of the blob centroids (Eq. 4) and midpoint of convex representation of binary objects (MP_convex_) were recorded via Eq. 5. In addition to the evaluation of the metrics derived from the test set, we reported the total failure rate (non-reasonable transformation) via manual inspection of the three images after estimated transformation via Eq. 6.

1 $$\:NCC=\:\frac{\sum\:{(I}_{A}-{\overline{I}}_{A})-({I}_{B}-{\overline{I}}_{B})}{\sqrt{\sum\:{{(I}_{A}-{\overline{I}}_{A})}^{2}\sum\:{({I}_{B}-{\overline{I}}_{B})}^{2}}}$$

2 $$\:OR=\:\frac{A\cap\:C}{\text{m}\text{i}\text{n}(A,C)}\times\:100$$

3 $$\:{OR}_{Convex}=\:\frac{{A}_{Convex}\cap\:{C}_{Convex}}{\text{m}\text{i}\text{n}({A}_{Convex},{C}_{Convex})}\times\:100$$

4 $$\:\varDelta\:MP=\:\frac{1}{\text{N}}\sum\:_{N=0}^{N}{|\overrightarrow{Centroid}}_{\left(A,N\right)}-{\overrightarrow{Centroid}}_{\left(C,N\right)}|$$

5 $$\begin{gathered}\Delta M{P_{Convex}} = \frac{1}{{\text{N}}}\mathop \sum \limits_{N = 0}^N |{\overrightarrow {Centroid} _{\left( {{A_{Convex}},N} \right)}} \hfill \\-\quad {\overrightarrow {Centroid} _{\left( {{C_{Convex}},N} \right)}}| \hfill \\ \end{gathered}$$

$$\:N\hspace{0.17em}=\hspace{0.17em}Number\:of\:binary\:objects$$

6 $$\:TF\:rate=\frac{TF}{{N}_{Fullset}}\times\:100$$

$$\:TF\:=\:Total\:failure\:of\:image\:registration;$$ $$\:{N}_{Fullset}\:=\:Number\:of\:registered\:images.$$

## Electronic supplementary material

Below is the link to the electronic supplementary material.

Supplementary Material 1: pdf: Parameterization of used image registration methods.

Supplementary Material 2: png: Flow chart of image reading multimodal image data and image registration pipeline. The python library for reading the multi-dimensional data is available in an open-access GitHub repository, https://github.com/halube/HyperKorReader.

Supplementary Material 3: pdf: Formulas of presented vegetation indices and calculated chlorophyll fluorescence parameters.

## Data Availability

The Python library for reading the multi-dimensional data is available in an open-access GitHub repository, https://github.com/halube/HyperKorReader.Supporting information contain mainly Methods section supporting figures and tables. The image datasets of the biological experiments are available from the corresponding author upon reasonable request.
