# Supplementary material for: Automated image registration of RGB, hyperspectral and chlorophyll fluorescence imaging data
Source: Plant Methods. 2024 Nov 17;20:175. doi: 10.1186/s13007-024-01296-y (PMC11572093; doi:10.1186/s13007-024-01296-y)
Supplement: Supplementary file 1 — Supplementary Material 1: pdf: Parameterization of used image registration methods. [file 13007_2024_1296_MOESM1_ESM.pdf]

Supplementary information 1: Parameters of the registration algorithms used

Method	References	Preprocessing	Modified parameter	Feature matching
Phase correlation	Ri & Fujimoto (2018)	—	—	—
Phase correlation + ECC	Ri & Fujimoto (2018)<br>Evangelidis&Psarakis (2008)	—	Max. Iteration: 10 000<br>Epsilon: 0.1	—
ORB	Rublee et al. (2011)	If no features found: Try: Histogram stretching	Max. Features: 1000	Brute Force Matcher (BF) using the Hamming distance
ORB + ECC	Rublee et al (2011)<br>Evangelidis&Psarakis (2008)	If no features found: Try: Histogram stretching	Max. Features: 1000<br>Max. Iteration: 10 000<br>Epsilon: 0.1	BF Matcher using the Hamming distance
ORB Parameter tuned	Rublee et al. (2011)<br>Lowe (2004)	Histogram stretching	Max. Features: 100 000	BF Matcher with K-Nearest Neighbors (k=2), distance filtering according to Lowe (2000) with ratio 0.8
ORB Parameter tuned +ECC	Rublee et al (2011)<br>Lowe (2004)<br>Evangelidis&Psarakis (2008)	Histogram stretching	Max. Features: 100 000<br>Max. Iteration: 10 000<br>Epsilon: 0.1	BF Matcher with K-Nearest Neighbors (k=2), filtering according to Lowe (2000) with ratio 0.8
NCC- adaptive approach	In this study	—	—	—
