# Supplementary figures and images for: Automated image registration of RGB, hyperspectral and chlorophyll fluorescence imaging data
Source: Plant Methods. 2024 Nov 17;20:175. doi: 10.1186/s13007-024-01296-y (PMC11572093; doi:10.1186/s13007-024-01296-y)

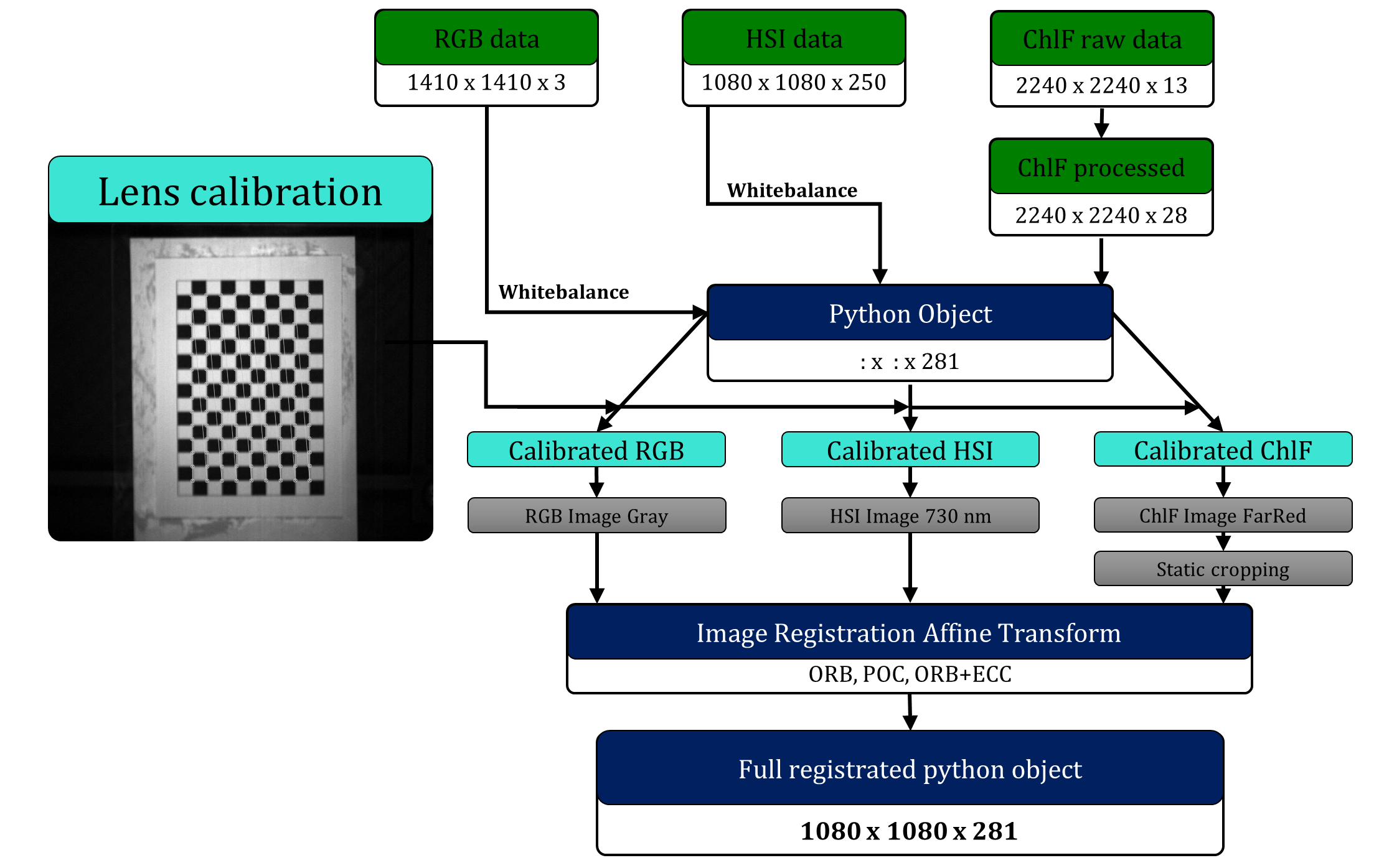

Supplement: Supplementary file 2 — Supplementary Material 2: png: Flow chart of image reading multimodal image data and image registration pipeline. The python library for reading the multi-dimensional data is available in an open-access GitHub repository, https://github.com/halube/HyperKorReader. [file 13007_2024_1296_MOESM2_ESM.png]
