# Supplementary material for: Automated image registration of RGB, hyperspectral and chlorophyll fluorescence imaging data
Source: Plant Methods. 2024 Nov 17;20:175. doi: 10.1186/s13007-024-01296-y (PMC11572093; doi:10.1186/s13007-024-01296-y)
Supplement: Supplementary file 3 — Supplementary Material 3: pdf: Formulas of presented vegetation indices and calculated chlorophyll fluorescence parameters. [file 13007_2024_1296_MOESM3_ESM.pdf]

Supplementary information 3: Calculated parameters/indices presented in the manuscript

Method	Definition	Formula with wavelength [nm]	References
$NDVI_{HSI}$	Normalized Difference Vegetation Index	$= \frac{\bar{R}_{740-760} - \bar{R}_{660-680}}{\bar{R}_{740-760} + \bar{R}_{660-680}}$	Rouse et al. (1974)
$ARI1_{HSI}$	Anthocyanin Reflectance Index	$= \frac{1}{\bar{R}_{548-532}} - \frac{1}{\bar{R}_{698-702}}$	Gitelson et al. 2001
CCI_{HSI}	Chlorophyll Carotinoid Index	$= \frac{\bar{R}_{530-534} - \bar{R}_{628-632}}{\bar{R}_{530-534} + \bar{R}_{628-632}}$	Gamon et al. 2016
$NDVI_{ChlF}$	Normalized Difference Vegetation Index	$= \frac{R_{730} - R_{660}}{R_{730} - R_{660}}$	Rouse et al. (1974)
F_v/F_m	Max. quantum efficiency of PSII photochemistry	$= \frac{F_m - F_0}{F_m}$	Genty et al. (1989)
NPQ	Nonphotochemical quenching	$= \frac{F_m - F_m{}'}{F_m{}'}$	Horton et al. (1996)

Note: $\bar{R}$ indicating mean intensity at pixel level over several wavelength
